# Exploring the Applicability of Physiological Monitoring to Manage Physical Fatigue in Firefighters

**DOI:** 10.3390/s23115127

**Published:** 2023-05-27

**Authors:** Denisse Bustos, Ricardo Cardoso, Diogo D. Carvalho, Joana Guedes, Mário Vaz, José Torres Costa, João Santos Baptista, Ricardo J. Fernandes

**Affiliations:** 1 Associated Laboratory for Energy, Transports and Aeronautics—LAETA (PROA), Faculty of Engineering, University of Porto, 4200-465 Porto, Portugal; ldbs@fe.up.pt (D.B.); jccg@fe.up.pt (J.G.); gmavaz@fe.up.pt (M.V.); jsbap@fe.up.pt (J.S.B.); 2 Centre of Research, Education, Innovation and Intervention in Sport—CIFI2D, Faculty of Sport, University of Porto, 4200-450 Porto, Portugal; up201200394@up.pt (R.C.); up201200415@up.pt (D.D.C.); 3 Porto Biomechanics Laboratory, Faculty of Sport, University of Porto, 4200-450 Porto, Portugal; 4 Associated Laboratory for Energy, Transports and Aeronautics—LAETA (PROA), Faculty of Medicine, University of Porto, 4200-319 Porto, Portugal; zecatoco@sapo.pt

**Keywords:** fatigue prediction, physiological signals, physiological variables, classification algorithms

## Abstract

Physical fatigue reduces productivity and quality of work while increasing the risk of injuries and accidents among safety-sensitive professionals. To prevent its adverse effects, researchers are developing automated assessment methods that, despite being highly accurate, require a comprehensive understanding of underlying mechanisms and variables’ contributions to determine their real-life applicability. This work aims to evaluate the performance variations of a previously developed four-level physical fatigue model when alternating its inputs to have a comprehensive view of the impact of each physiological variable on the model’s functioning. Data from heart rate, breathing rate, core temperature and personal characteristics from 24 firefighters during an incremental running protocol were used to develop the physical fatigue model based on an XGBoosted tree classifier. The model was trained 11 times with different input combinations resulting from alternating four groups of features. Performance measures from each case showed that heart rate is the most relevant signal for estimating physical fatigue. Breathing rate and core temperature enhanced the model when combined with heart rate but showed poor performance individually. Overall, this study highlights the advantage of using more than one physiological measure for improving physical fatigue modelling. The findings can contribute to variables and sensor selection in occupational applications and as the foundation for further field research.

## 1. Introduction

Fatigue is a complex and multidimensional condition, often described as a decrement in mental or physical performance caused by factors such as physical exertion, sleep deprivation, circadian rhythm disruption or illness [1,2]. In addition to being a physiological response of the human body, possibly preventing its overload, fatigue can be a symptom associated with several diseases and health conditions [1]. According to the type of load, it has been classified as physical (resulting from corporal exertion and leading to a decrease in overall performance) and mental (resulting from sustained cognitive activity and leading to a reduction in intellectual and behavioural performance) [3,4]. Physical fatigue is a common issue among many occupational groups, making its quantification of crucial relevance in occupational health and safety [2,5,6].

Firefighters, along with other high-risk professionals (e.g., military and law enforcement officers), face situations of extreme danger and physiological stress caused by high-demand tasks, long duty periods, extreme environmental conditions, and sleep deprivation, all of which aggravate the risk of physical overload, injury or illness [7,8]. Their roles involve a high level of physical exertion with tremendous demands on the cardiorespiratory system [9]. Firefighters must respond to and suppress fires and deal with medical emergencies, adverse roadways, and hazardous material events. Inherent to the nature of their profession, they are at an increased risk from various physical and psychological workplace stressors and increased potential for injury and impairing their overall performance and wellbeing [10]. Vast amounts of information in the literature have shown firefighting duties as a civilian occupation with high variability of exposure to physical stress and risks [11]. The ability to monitor their physiological response during daily activities may help protect their health [7,12].

Continuous physiological assessment can improve safety monitoring and work–rest planning to maximise effective and safe performance among firefighters and other high-risk professions [13]. To better understand physical exertion as the precursor of physical fatigue and other related health impairments, research efforts have increasingly recurred to physiological variables to monitor its intensity and be able to manage its effects proactively. With advances in wearable sensor technology, it is becoming easier to noninvasively retrieve physiological signals in real time, with their combined analysis leading to more accurate assessments [3,14]. Current physical fatigue quantification approaches based on physiological signals involve mainly supervised learning algorithms (e.g., support-vector machines [15,16], random forests [17,18], boosted trees [19]) addressing fatigue as a binary classification problem [17,18], or considering three [20] or four [19] fatigue levels. Although less common, indices calculated from the monitored variables [21] and statistical modelling techniques [22] have also been explored for this goal. Among occupational groups, supervised machine learning algorithms for fatigue prediction during construction [19], manufacturing tasks [23], and some within firefighters [24,25] are the most recurrent in the literature.

Overall, recent studies are based on physiology and principled computational techniques to develop algorithms that allow sensors to be used for individualised physical fatigue assessment, making wearable sensor data useful and actionable in real-time applications [3,13]. Although these studies have shown that the best results come from assessing multiple physiological measures, problems such as the unfeasibility of measuring several variables, compromised data quality, or data loss during specific periods can occur in occupational settings. Therefore, they have to be taken into account to guarantee that, despite them, the system using the algorithm still provides reliable outcomes. Hence, besides evaluating their prediction performance, the interpretability and usability of these approaches need to be further explored. For this, the contribution of features and sensors has to be comprehensively analysed to facilitate decision-making and variable selection for real-life occupational settings while determining the model’s performance when removing one or another of the model’s features. A four-level physical fatigue prediction model was developed using a multivariable physiological method and supervised machine learning algorithms [26], and the current work aims to analyse the influence of each physiological measure on its performance by comparing the output variations using different combinations of physiological signals.

## 2. Materials and Methods

### 2.1. Experimental Design

Data from 24 active volunteer firefighters (18 men) were collected to develop the model (Table 1 summarises their main anthropometric characteristics). Participants had no history of cardiopulmonary or intestinal diseases and did not report any musculoskeletal disorders. All of them provided written informed consent before data collection and the Ethics Committee of the University of Porto approved the experimental procedures in accordance with the Declaration of Helsinki (Report 106/CEUP/2021). The experimental protocol consisted of incremental intermittent running during seven 4 min stages with 1 km/h increments and 30 s resting intervals between stages to voluntary exhaustion [26]. It was performed on a treadmill (T2100 treadmill; GE, Boston, MA, USA) [27,28] inside a climatic chamber (FITOCLIMA 25000 EC20; Aralab, Rio de Mouro, Portugal) [29] with an air temperature of 24 °C and relative humidity of 50%. The initial velocity was defined according to each volunteer’s experience and capacity [28,30].

During the incremental protocol, breath-by-breath respiratory gas exchange was assessed using a portable telemetric gas analyser (Cosmed K5; Cosmed, Rome, Italy) placed near the participants’ body centre of mass [28,30]. Heart rate was recorded at rest and every 5 s using a Garmin Sensor that telemetrically sent the data to the K5 equipment. Intra-abdominal core temperature was retrieved every 15 s with telemetric ingestible thermometer pills (e-Celsius Performance capsule; BodyCAP, Hérouville-Saint-Clair, France) ingested by the participants 6 h before the tests [31]. Participants’ perceived exertion was asked at the end of every 4 min step using the 6–20 Borg scale [28].

### 2.2. Data Processing and Modelling

#### 2.2.1. Physiological Variables

Signals from heart rate, breathing rate, and core temperature were used as the main variables for modelling since they can be obtained using sensors feasible to be used within extreme environments, allowing mobility, uninterrupted monitoring, and comfort [32,33,34]. Heart rate is the most easily measured vital sign, being widely used to measure physical exertion and overload [18] and to determine the physiological demands and workload of firefighting activities [35,36]. Considering the importance of respiratory frequency as an indicator of physical exertion during exercise [37] and its inclusion in recent fatigue quantification approaches [38,39], it was also integrated to investigate its usefulness in improving physical fatigue modelling among firefighters. Furthermore, core body temperature was considered since it has also been found to be strongly related to time to exhaustion during exercise [40], and recent reviews have addressed its applicability within some occupational environments [6,41].

#### 2.2.2. Data Preprocessing

Retrieved data were initially edited to remove noisy records resulting from talking, coughing, or any other signal interruption [26,28,30]. Then, values varying more than three standard deviations were eliminated and the remaining records were smoothed using a moving average filter [27,28,30]. Borg’s levels 6–11 were combined to represent low physical fatigue, 12–14 were grouped as moderate physical fatigue, 15–16 were classified as heavy, and values from 17–20 were considered severe physical fatigue status [19,42,43].

#### 2.2.3. Physical Fatigue Classification Model

Preprocessed data were synchronised using 1 min intervals and features including mean, maximum, minimum, and baseline values were calculated from heart rate, breathing rate, and core temperature signals. These 12 features were combined with the age-predicted maximum heart rate (220-age) [30], the percentage of the age-predicted maximum heart rate, and personal characteristics (i.e., age, gender, weight, height, fat mass, fat-free mass, and body mass index) as inputs for modelling. The simplified levels from Borg’s scale (i.e., low, moderate, heavy, and severe) were used to classify physical fatigue [3,19,26]. The dataset, comprising a total of 750 sets of 21 features (seven from personal variables, six from heart rate, four from breathing rate, and four from core temperature), and the corresponding fatigue levels (283 records with low labels, 140 of moderate, 167 of heavy, and 160 corresponding to severe level), were normalised and fed into machine learning algorithms.

Previously, various classification algorithms were tested with different parameter configurations and using three cross-validation procedures [26]. K-nearest neighbours, bagged trees, boosted trees (gradient-boosted trees, XGBoosted trees and RUSBoosted trees), random forests, support vector machines with different kernel functions (linear, quadratic, cubic, and Gaussian) and artificial neural networks were evaluated since they have been successfully used for occupational purposes involving physiological signals [19,26,43]. The best performance metrics were obtained using the XGBoosted trees, set to use 500 estimators, a maximum depth of individual regression estimators of five and a learning rate of 0.1, validated using group cross-validation with 24 splits. This cross-validation method (evaluated previously along with 10-fold and stratified cross-validation methods) gave the most representative results since it evaluated the performance of the model in each individual, avoiding potential overfitting from using the same subject’s datapoints to train and test the model [26].

To compare the effectiveness of physiological variables to classify the four physical fatigue levels, the performance variations, while restricting the number of features, were evaluated. For this, the model was trained 11 times, with one considering all features, four removing one group of features each time (i.e., personal characteristics, heart rate, breathing rate, and core temperature), three considering features from one physiological signal, and personal characteristics and three including only features from one physiological signal. The models’ performance was determined according to four performance measures: accuracy, precision, recall, and F1-score [44], which were averaged from the 24 splits.

## 3. Results

**Figure 1:**
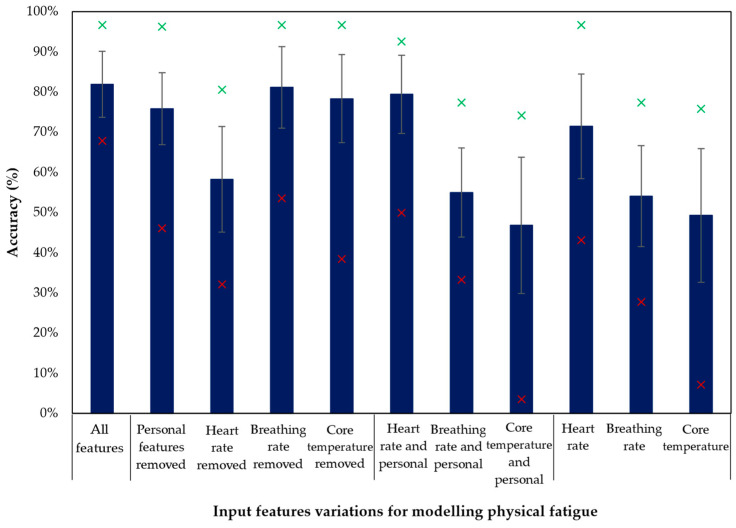
Overall accuracies using the 11 assessed combinations of features. The error bars indicate the standard deviations, and the green and red markers point to the maximum and minimum reached accuracies for each case.

The predictive accuracies (based on group cross-validation with splits according to the number of participants) of training the XGBoosted trees model using the 11 feature combinations are described in Figure 1, with the variability of individual participants’ results within each scenario being also illustrated. As expected, the highest accuracy and lowest variability were reported by using all features. Nevertheless, results also show that accuracies are relatively high for six models (above 75%), but the gaps between the maximum and minimum values go up to 50% in some cases (e.g., using only heart rate features). The mean (and standard deviation) for the other performance measures with the number of features selected in each model are reported in Table 2.

**Figure 2:**
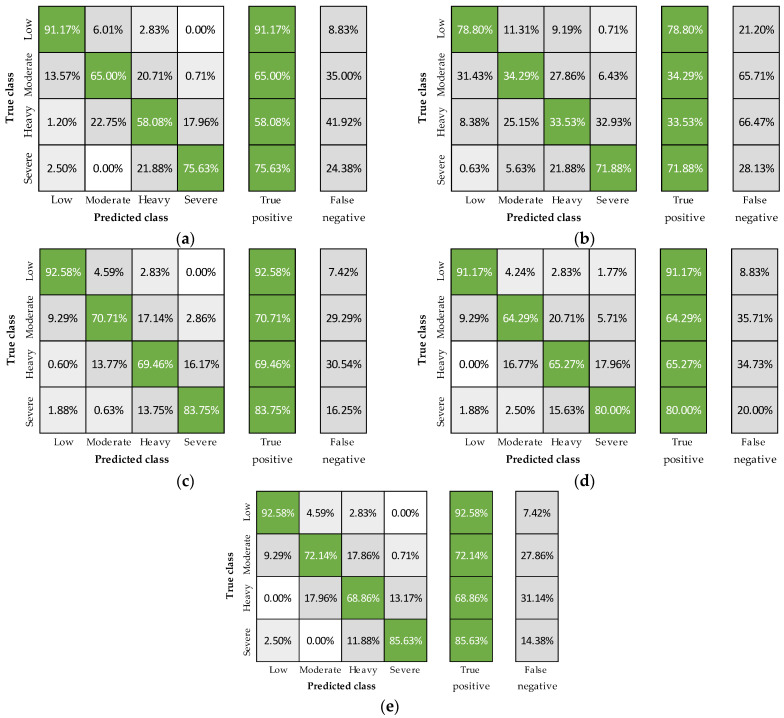
Confusion matrices excluding personal (**a**), heart rate (**b**), breathing rate (**c**), core temperature (**d**) and using all features (**e**).

In Figure 2, the classification accuracies obtained in the four physical fatigue levels are illustrated for five scenarios, four excluding one group of features at a time and one including all the features. Although personal features are fixed values that are generally easy to obtain, they were removed from the model to determine its behaviour solely based on the sensor signals. By excluding these features, the overall accuracy dropped to 75%, with the moderate and heavy levels being the most affected. Results also evidenced the critical importance of heart rate, with its exclusion causing substantial decreases in the moderate and heavy correct predictions. Furthermore, when removing core temperature or breathing rate, it was observed that the first might be a better alternative to improve physical fatigue assessment.

**Figure 3:**
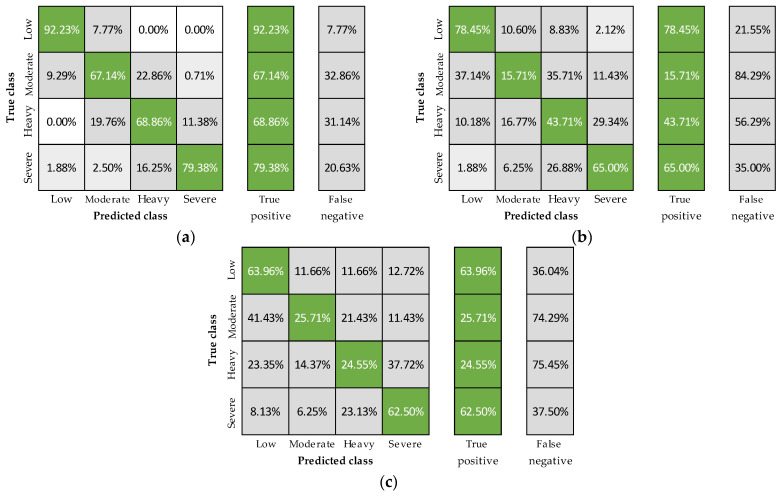
Confusion matrices including heart rate and personal (**a**) breathing rate and personal (**b**) and core temperature and personal features (**c**).

**Figure 4:**
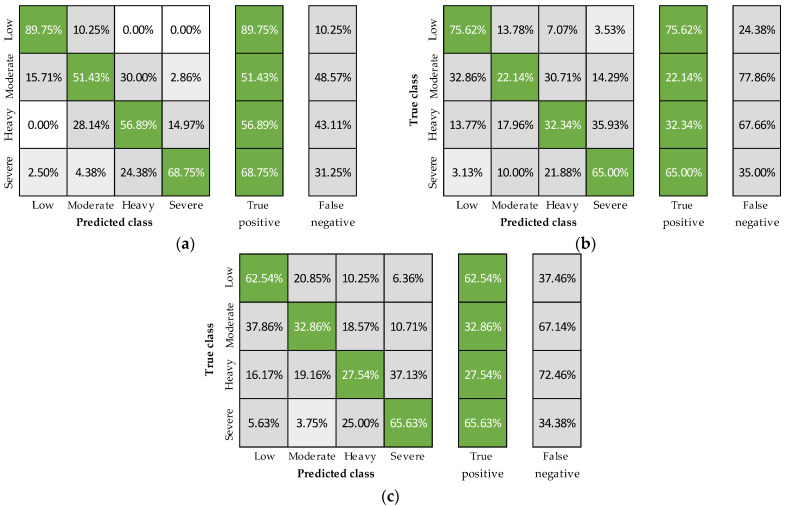
Confusion matrices including heart rate (**a**), breathing rate (**b**), and core temperature features (**c**).

To determine the independent ability of each physiological signal to predict fatigue, the XGBoosted tree model was trained six more times with the 24-fold group-cross-validation and using the features extracted from a single sensor with and without personal features (Figure 3 and Figure 4). Including personal features, accuracies were 80, 56, and 48%, using heart rate, breathing rate, and core temperature, respectively (Figure 1), with the best predictions registered in the low and severe fatigue levels (Figure 3). On the other hand, removing the personal features resulted in classification accuracies of 71, 54, and 50% for heart rate, breathing rate, and core temperature, respectively (Figure 1). The decreases observed using heart rate and breathing rate (a and b panels from Figure 3 compared to a and b panels from Figure 4) show that these features are essential to account for the physiological differences among subjects. In contrast, core temperature’s performance evidenced that this signal improves fatigue detection when combined with other physiological variables (Figure 2) but has a poor prediction performance independently.

## 4. Discussion

Workers’ physical fatigue is an important safety concern in high-risk professions and continuously monitoring it is essential to protect their health and wellbeing. The current study results show that combining cardiorespiratory and thermoregulatory measures is an accurate alternative to monitor and model physical fatigue for occupational purposes. Although some of the studied variables proved to be more effective than others, outcomes point out that more than one of the studied physiological variables are needed to reliably predict four fatigue levels. Monitoring changes in heart rate and core temperature can provide more useful information than monitoring the latter with breathing rate. Therefore, when it would be impossible to monitor all variables simultaneously, managers can use these results to decide between them and avoid a considerable decrease in overall accuracy in fatigue monitoring.

The machine learning domain contains a wide variety of models based on learning ability, adaptiveness, complexity and scalability [45]. From them, deep learning algorithms have gained relevance within the health and safety area, addressing important issues such as fall detection [46,47], prediction of diseases [48,49], or working-site safety improvement [50,51]. Most of these approaches are very effective for producing high-quality results, robust to signal noise and involve automated feature extraction [52,53,54]. However, they also require massive data and high processing power while lacking interpretability [45]. Despite the high performance that machine learning models can reach, their real-life applicability remains limited if a comprehensive understanding of the underlying model self-arranged through the training algorithm is not extracted [55]. Consequently, despite their advantages, they have not been used in multilevel fatigue quantification approaches, being only included in binary fatigue prediction models (recurrent neural networks were used in [56]), mental fatigue (multilayer neural networks were applied in [57]), and drowsiness (convolutional neural networks were used in [58]) detection with accuracies around 70%, which is under the accuracy reached by other techniques [3]. Overcoming data and interpretability constraints, supervised learning models have been predominant for fatigue quantification models, with decision trees, support-vector machines and random forests being the most common [3,59]. Therefore, different alternatives of these supervised algorithms were evaluated previously [26] with the XGBoost classifier determined to have the best performance.

The current approach intends to overcome the “black box” nature of machine learning algorithms by analysing different input combinations that can guide model evaluation and application while uncovering potential erratic behaviour stemming from overfitting or insufficient training dataset size [17,60]. The 11 input combinations assessed in this study helped to observe the severity of change in the model’s output resulting from the change of given inputs, providing insight into the influence of variables on outputs. Although this information can be, to an extent, extracted by plotting the model’s feature importances, it does not allow us to see the proportion of change in the output by excluding one or other input. In addition, it does not provide a comprehensive view of reached accuracies given the different combinations that, for occupational applications, would be a fundamental tool to make informed decisions on sensor selection and placement. As a result, the current study’s framework contributes to occupational fatigue monitoring by affirming the XGBoosted tree classifier’s usefulness for physical fatigue prediction in firefighters and providing an analytical approach in which every signal role is explored and determined.

**Figure 5:**
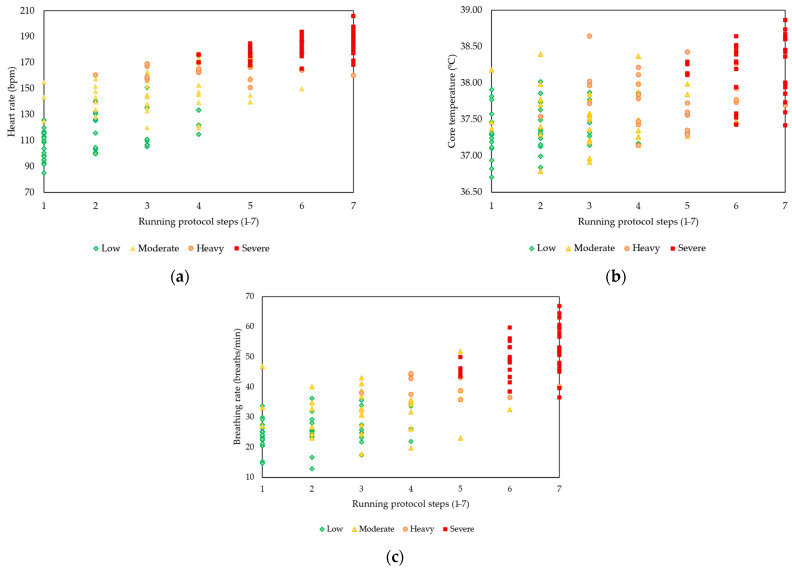
Relationship between heart rate, core temperature, and breathing rate mean values and the physical fatigue scale through the running protocol ((**a**–**c**) panels, respectively).

Given the inter- and intra-subject variability in human physiological states and responses, individualised monitoring has become key in fatigue quantification approaches [3,34]. It helps overcome the inconvenience and subjectivity of previous methods based on unrealistic thresholds established for standard individual profiles, subjective scales or questionnaires [3]. In the evaluated dataset, the variability among participants’ responses could be observed in the three physiological variables under the different physical fatigue levels (Figure 5). For example, at the low physical fatigue level, the participants had a range of heart rate, core temperature and breathing rate of 85 to 151 bpm, 13 to 36 bpm and 36.7 to 38.0 °C (respectively). Interestingly, a decrease in interindividual differences could be observed for core temperature with the increase in exertion levels (Figure 5c). This behaviour might help explain the increase in accuracy with an increase in physical fatigue levels (Figure 2e) from heavy to severe intensities. These outcomes confirm the need for the individualised assessment of physiological signals and the practicality of this approach to translate physiological responses into simple and interpretable information.

Based on the results described in Figure 2, Figure 3 and Figure 4, some remarks can be outlined. First, the best-obtained accuracies were with all features (Figure 2e), confirming the multifactorial nature of fatigue, benefiting from the assessment of various signals to determine its occurrence and intensity. Correspondingly, cases excluding one group of variables (Figure 2a–d) showed mostly higher classification accuracies than considering only one physiological signal with or without personal features (Figure 3 and Figure 4, respectively). Nevertheless, the inclusion of heart rate consistently led to better outcomes, describing its critical relevance for physical fatigue monitoring. As for breathing rate and core temperature signals, they helped increase the overall classification accuracy and reduce variability when combined with heart rate and showed poor performance when used independently. However, despite the demonstrated usefulness of heart rate in the current study and available literature [14,38], multiple factors (e.g., stress, panic or anxiety) can alter this signal without a physical fatigue condition [38,61,62]. Thus, including the other variables can undoubtedly reduce potential false positives in its detection while narrowing the gaps among individual accuracies. Finally, the best results were registered in the low and severe levels for all cases, confirming the model’s capacity to predict extreme scenarios, which is crucial for occupational applications.

In general, the current results are aligned with recent related studies in which multiple signals have been retrieved (almost all including heart rate [19,38,43]). However, the importance given to features from this variable differed among them. Although it is impossible to directly compare the current with other studies because of the diverse physiological signals, dataset sizes, fatiguing tasks and samples, some observations can be made. Similar to the current work, a study used a four-level exertion scale derived from the 6–20 Borg scale as labels and developed a fatigue classification model based on a decision tree classifier [19]. They used data from electroencephalography, various infrared temperature sensors on the face and heart rates from 12 construction workers during simulated construction tasks. Their results showed that thermoregulation and heart rate signals produce better results than using each variable separately. However, their further analysis evidenced that skin temperature is more determinant for physical exertion prediction than heart rate. Hence, the importance of heart rate for determining different fatigue levels can be confirmed and future studies should be directed to corroborate its primary importance in real-life settings.

Alternatively, while evaluating construction workers, motion and heart rate signals were monitored during fatiguing manufacturing tasks, particularly simulated manual material handling and supply pick up and insertion [17]. They used the RPE scale, but unlike the previous [19] and the current study, it was considered RPE ≤ 13 as a cut-off for fatigue detection. Their results described that motion-derived features were more relevant than heart rate when evaluating the first task and this latter variable was more important for the second. Furthermore, another study used a similar approach to assessing firefighters’ responses during training by monitoring core body temperature and heart rate using the Borg scale simplified to a two-point scale to classify low (6–10) and high strains (15–20) [24]. While both signals were needed for achieving the best predictions, the temperature was the variable to which more importance was given in the model. Regarding measuring breathing rate, it has been included in various studies [22,43], but its importance in their developed models was not explored. Despite the lack of consensus regarding the most important physiological signal, related studies help confirm the need for integrated assessments from multiple physiological signals to predict physical fatigue reliably.

There are some limitations that may impact the interpretation of the obtained results (such as the participants’ characteristics and the fact that they were evaluated only during running). Even though the step protocol allowed for the capture of the increments in physiological responses until maximal aerobic exertion, different tasks should be studied for a real-life application of a fatigue monitoring system. Furthermore, given that environmental factors, especially temperature and humidity, affect fatigue substantially, future studies should evaluate how this model performs in different environmental conditions. These new studies will allow not only to validate the model’s performance but also to assess the sensors’ functioning and functionality and the quality of data obtained under those conditions. Finally, comparing the current results with the available literature, it was evidenced that the importance of physiological signals varies among occupational groups and fatiguing tasks, meaning that researchers and practitioners should consider this finding when developing models for detecting and managing fatigue in other settings.

## 5. Conclusions

The objective of the current study was to investigate the usefulness of monitoring multiple physiological variables changes to predict the level of physical fatigue by analysing their individual and combined predicting capability. The evaluation of 11 input combinations demonstrated the capability of using an integrated modelling approach for managing physical fatigue. The results described that the prediction performance considerably improved using various signals compared to using only one. In this regard, the outcomes demonstrated that monitoring heart rate can provide more valuable information than monitoring core temperature or breathing rate for physical fatigue assessment. However, their combined use leads to better classification accuracy, reducing the gaps in the accuracies among the four considered levels and the potential false positives from heart rate univariate monitoring. These findings can be used to facilitate decision-making on sensor selection. Future studies must be directed to evaluate the model’s capability to detect the gradual increment in fatigue in multiple occupationally relevant settings.

## Figures and Tables

**Table 1:** Firefighters’ age and general anthropometric characteristics.

	Mean	SD	Min	Max
**Age (years)**	33.08	9.73	19.00	51.00
**Weight (kg)**	75.98	10.79	58.90	104.20
**Height (cm)**	173.10	8.12	150.20	189.00
**Fat mass (%)**	22.69	10.85	7.32	50.12

**Table 2:** Physical fatigue classification performance metrics mean (and the corresponding standard deviation) for the different combinations of features.

Input Features Variations	Features (n)	Precision	Recall	F1-Score
All features	21	82.25 (10.93)	82.24 (10.13)	82.06 (10.34)
Personal variables removed	12	75.67 (14.05)	75.60 (12.84)	75.84 (13.16)
Heart rate features removed	15	58.39 (18.81)	59.09 (21.34)	58.43 (19.97)
Breathing rate features removed	17	81.52 (10.14)	81.63 (10.09)	81.39 (9.71)
Core temperature features removed	17	78.23 (12.12)	77.82 (11.55)	78.09 (11.92)
Heart rate and personal features	13	79.90 (14.02)	79.44 (10.27)	79.89 (11.99)
Breathing rate and personal features	11	54.21 (20.45)	56.08 (24.04)	54.96 (22.49)
Core temperature and personal features	11	46.41 (13.01)	47.80 (19.10)	46.69 (15.59)
Only heart rate features	6	71.80 (18.73)	70.89 (15.47)	71.14 (16.69)
Only breathing rate features	4	52.50 (18.62)	53.78 (22.93)	52.94 (20.73)
Only core temperature features	4	49.91 (15.07)	50.25 (17.34)	49.36 (15.26)

## Data Availability

Data is contained within the article.
